# Diagnosis of Human Cytomegalovirus Drug Resistance Mutations in Solid Organ Transplant Recipients—A Review

**DOI:** 10.3390/diagnostics14020203

**Published:** 2024-01-18

**Authors:** Madain S. Alsanea, Ahmed A. Al-Qahtani, Reem S. Almaghrabi, Maha A. AlAbdulkareem, Basma M. Alahideb, Dalia Obeid, Feda A. Alsuwairi, Fatimah S. Alhamlan

**Affiliations:** 1 Department of Infection and Immunity, King Faisal Specialist Hospital and Research Center, Riyadh 11564, Saudi Arabia; madainsaleh2@gmail.com (M.S.A.); aqahtani@kfshrc.edu.sa (A.A.A.-Q.); moalabdulkareem@kfshrc.edu.sa (M.A.A.); balahideb@kfshrc.edu.sa (B.M.A.); obeiddx@gmail.com (D.O.); falsuwairi@kfshrc.edu.sa (F.A.A.); 2 College of Medicine, Alfaisal University, Riyadh 11533, Saudi Arabia; 3 Organ Transplant Center of Excellence, King Faisal Specialist Hospital and Research Center, Riyadh 11564, Saudi Arabia; ramaghrabi@kfshrc.edu.sa; 4 Department of Pathology and Laboratory Medicine, King Faisal Specialist Hospital and Research Center, Riyadh 11564, Saudi Arabia

**Keywords:** cytomegalovirus, CMV, organ transplant, diagnosis, Sanger sequencing, NGS, drug resistance

## Abstract

Human cytomegalovirus (HCMV) infection may be asymptomatic in healthy individuals but can cause severe complications in immunocompromised patients, including transplant recipients. Breakthrough and drug-resistant HCMV infections in such patients are major concerns. Clinicians are first challenged to accurately diagnose HCMV infection and then to identify the most effective antiviral drug and determine when to initiate therapy, alter drug dosage, or switch medication. This review critically examines HCMV diagnostics approaches, particularly for immunocompromised patients, and the development of genotypic techniques to rapidly diagnose drug resistance mutations. The current standard method to identify prevalent and well-known resistance mutations involves polymerase chain reaction amplification of UL97, UL54, and UL56 gene regions, followed by Sanger sequencing. This method can confirm clinical suspicion of drug resistance as well as determine the level of drug resistance and range of cross-resistance with other drugs. Despite the effectiveness of this approach, there remains an urgent need for more rapid and point-of-care HCMV diagnosis, allowing for timely lifesaving intervention.

## 1. Introduction

Human cytomegalovirus (HCMV) is a widespread, double-stranded DNA virus that establishes lifelong latency after primary infection [1,2,3,4]. The outcomes vary from asymptomatic infection to life-threatening or fatal disease, depending on the patient’s immune status [5]. In healthy individuals, HCMV infection is often mild or asymptomatic [3,6]. By contrast, it is highly pathogenic among immunocompromised patients, such as solid organ transplant (SOT) recipients and individuals with allogeneic hematopoietic stem cell transplantation (HSCT), causing life-threatening disease [2,3,6,7,8,9]. HCMV disease can manifest as hepatitis, nephritis, pneumonitis, myocarditis, and pancreatitis, consistent with the transplanted organ [8,9].

HCMV-associated disease represents a main cause of morbidity and mortality among SOT recipients without antiviral intervention within the first three months after transplantation [4,7]. International guidelines recommend administering antiviral therapy guided by personalized resolution of clinical symptoms and serial measures of patient viral load [3,8,10]. The rate of rise in the viral load is an important marker of CMV disease risk, as there is an association between the pace of rise in CMV load and increasing risk of CMV disease [4]. However, several approved antiviral drugs exist, including ganciclovir, the prodrug valganciclovir, foscarnet (FOS), and cidofovir [11,12,13]. Recently, the U.S. Food and Drug Administration (FDA) approved letermovir for use in CMV infection prophylaxis for CMV-seropositive HSCT) recipients [8,9,14,15]. However, the success of anti-HCMV treatment is influenced by many factors, such as the diagnostic method, the severity of immunosuppression, the concentration of the administered antiviral drug, and the viral strain’s susceptibility to the drug [2,7]. 

Drug-resistant mutant viruses often appear after the prolonged exposure of HCMV to antiviral drugs [11]. Therefore, it is fundamentally important to understand the development of resistance mutations through the characterization of drug resistance in clinical specimens from patients receiving antiviral therapy [5]. Currently, sequencing portions of the UL97, UL54, and UL56 genes, where common resistance mutations have been described, using Sanger sequencing is the most accessible and scalable method for routine genotyping [15]. However, next-generation sequencing (NGS) makes data mining easier and allows for the unbiased detection of low frequency events that Sanger sequencing typically cannot detect. Further developments may make it easier to identify known resistance markers earlier in patients who are at risk or to identify novel mutations that are currently inaccessible through routine testing [16,17]. Although advances in molecular virology and improvements in diagnostic methods and treatment options have greatly increased our understanding of and ability to treat HCMV, there are still numerous unanswered questions. In this review, we aim to provide a concise overview of HCMV infection in transplant recipients and to discuss diagnostic methods for resistance mutations.

## 2. HCMV Infection 

HCMV, also known as human herpesvirus 5, is ubiquitous worldwide, with a seroprevalence estimated at 90% in the Eastern Mediterranean region and 83% globally [6,9]. Although most HCMV infections in immunocompetent individuals are benign and self-limiting, they are an important cause of morbidity and mortality in individuals with compromised or immature immune systems, including transplant recipients [7,8,18]. In solid organ transplant (SOT) recipients, the risk of acquiring HCMV disease depends on many factors, including the serostatus of the donor and recipient, the type of organ transplanted, and the intensity and type of immunosuppressive therapy used [4,5,8,18,19]. A recent study reported that the percentage of HCMV infection among the total studied recipients was highest in heart transplantation, followed by multi-organ, pancreas, kidney, lung, and liver transplantation [20]. Moreover, HCMV infection has been linked to higher mortality, an increased risk of acute rejection and graft failure, and higher costs for inpatient care, readmissions, and hospital expenses [13]. 

HCMV infection is defined as the detection of the HCMV antigen or nucleic acid and the isolation of the virus from a patient’s sample. HCMV replication may be used at certain times as a substitute for determining HCMV infection, as it provides evidence of virus multiplication [21]. Becoming infected with the virus for the first time, known as the primary infection, may establish latency and thus play a role in the development of drug resistance [3,5,21]. HCMV establishes lifelong latency in the majority of infected persons, who can experience recurrent infection from reactivation of this latent virus (endogenous) or reinfection with a strain distinct from that which caused the initial infection (exogenous) [3,5,6,18,21]. HCMV reactivation is linked to longer stays in the hospital and intensive care unit, a higher risk of infection, a longer time spent requiring mechanical ventilation, and twice the mortality rate of severely sick patients [10]. Therefore, for patients at risk for severe HCMV illness, diagnosis, monitoring of active HCMV infection, and in many circumstances, long-term HCMV antiviral therapy, are lifesaving procedures [7].

### 2.1. Effects of HCMV Infection 

The effects of CMV infection associated with active viral replication in organ recipients may be direct or indirect. Fever, neutropenia syndrome, and end-organ disease, such as pneumonitis, hepatitis, nephrites, pancreatic, and encephalitis, are among the main signs of the direct effects [2,12,17,18]. Increasing immunosuppression by immunomodulatory molecules produced in reaction to viral infection of the body increasing the chance of other opportunistic infections is considered an indirect effect [1,2,3,12,22]. In immunocompromised patients, CMV pneumonia is a common clinical manifestation of the illness [12,21,22]. The indirect effects of HCMV may result in more morbidity overall than is presently attributed to end-organ disease [3].

### 2.2. Risk Factors 

CMV infection and disease occurrence differs by a number of risk factors, including allograft rejection, the posttransplant immunosuppressive protocol, and the serostatuses of the donor and recipient [1,2,3,8,18,23,24]. Allograft rejection and CMV infection have a bidirectional relationship [8]. A recent study reported that in a matched cohort, CMV infection was an independent risk factor (hazard ratio, 1.93; *p* = 0.012) for survival of the transplanted liver [25]. Rejection of an allograft induces a pro-inflammatory environment that can reactivate CMV. In addition, therapy for allograft rejection significantly reduces the body’s capacity to mount an immune response to stop viral replication. By contrast, CMV upregulates antigens, which causes alloreactivity and facilitates allograft rejection [8]. Furthermore, it has been shown that some immunosuppressive medications themselves may lead to an active systemic viral infection, whereas other immunosuppressive medications may reduce the likelihood of infection [5,7,8,26]. The most crucial pretransplant risk factor for CMV disease are the serological statuses of the donor (D) and recipient (R) [12,13,24]. 

Patients who receive an SOT from a CMV-seropositive donor (D+) have the highest risk of developing CMV infection and disease, whereas R−/D− recipients have the lowest risk [1,8,13,14,18,24]. Compared with R-/D+ individuals, R+/D+ grafts and recipients have a smaller survival period [14]. However, patients with grafts or allografts who are in subgroups at risk of active HCMV infection continue to have a reduced survival rate, according to clinical cohorts [3]. A previous study found that R−/D+ patients, which represented 70% of the samples, had an odds ratio of 5.8 (95% confidence interval, 1.6–21.2) for antiviral resistance development compared with seropositive recipients. In addition, the viral load was higher in the R−/D+ group (*p* = 00.12). Compared with that in R+ patients, the development of antiviral resistance was more frequent in patients with high viral loads (*p* = 0.03) [27]. 

### 2.3. HCMV Reactivation in Immunocompromised Patients 

**Figure 1:**
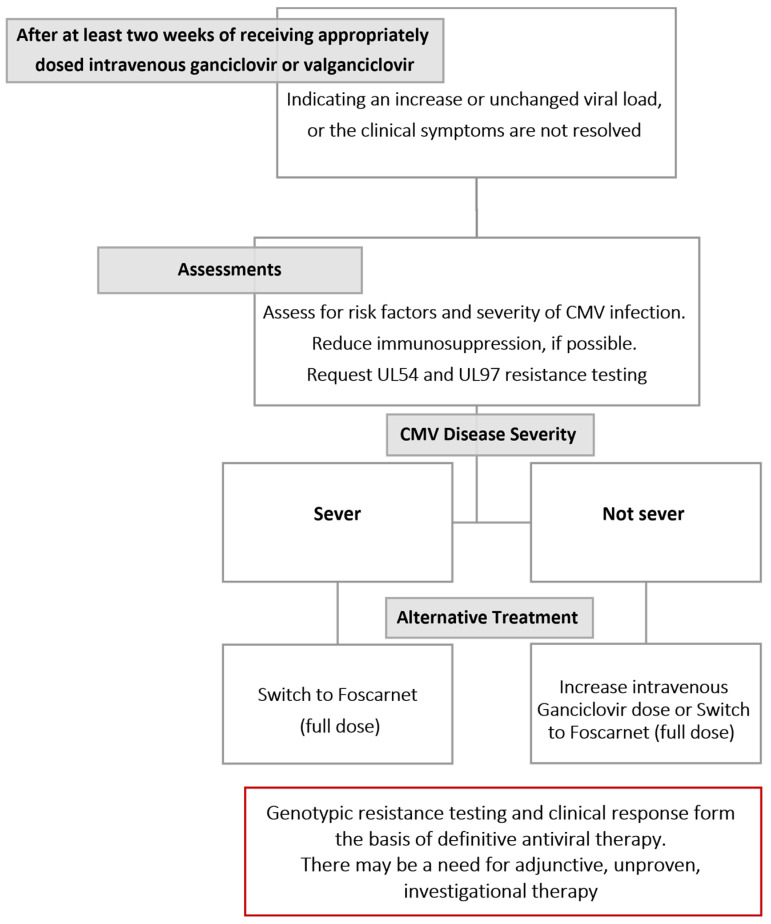
Algorithm published by Disease Community of Practice guidelines for evaluation and management of resistant and refractory cytomegalovirus infection and disease [29].

Reactivation in immunocompromised patients may be asymptomatic, a condition known as “CMV infection”, or may be symptomatic, defined as “CMV disease”. CMV infection is associated with increased risk of graft failure in SOT and high morbidity and mortality. However, there are two subdivisions of CMV disease: “CMV syndrome”, when a patient does not have organ disease but has malaise, fever, leukopenia or thrombocytopenia; and “tissue-invasive disease”, when organs are involved [5,10,18,28,29]. In patients with a liver transplant, 60% of CMV disease has been characterized as CMV syndrome, while tissue-invasive disease is recognized in 11–17% [1]. Moreover, there is an association between CMV disease and viral load. While there is considerable overlap between these categories, CMV viral syndrome is generally associated with an intermediate range of CMV viral load values, whereas higher values are linked to tissue-invasive disease; lower CMV viral load values are observed with asymptomatic CMV infection [4]. The Infectious Disease Community of Practice (IDCOP) published cytomegalovirus in the solid organ transplant recipients guidelines in 2019, which has an algorithm for evaluating and managing cytomegalovirus infections and diseases that are refractory and resistant, as illustrated in Figure 1 [29]. 

## 3. Best Practices for Clinical HCMV Detection in SOT Recipients 

The CMV serostatus of the donor (D) and recipient (R) before transplantation is a critical indicator of CMV risk after transplantation. Thus, the third international consensus guidelines on the management of CMV in SOT and the American Society of Transplantation Infectious Diseases Community of Practice guidelines recommend using serological tests with high sensitivity and specificity [29,30]. Serological assays measuring IgM or IgG and IgM combined have lower specificities and may result in false-positive results; therefore, a test detecting CMV-specific IgG should be used. Furthermore, when D- or R- cases are identified during the pre-transplant evaluation, the serological tests should be repeated at the time of the transplantation [28,29]. However, it is not recommended to use serology tests for the diagnosis of CMV infection after SOT because the capacity of SOT recipients to produce a significant antibody response is compromised due to their required immunosuppression. In addition, patients who receive blood products at the time of or following transplantation may experience false-positive results from passively transmitted antibodies [29]. 

Following transplantation, early detection and quantification of the infectious agent are vital for the effective management of antiviral drug resistance [28,30,31]. Nucleic acid amplification tests (NATs) in general and quantitative nucleic acid amplification tests (QNATs) in particular are the preferred methods for diagnosing CMV infection, deciding on preemptive strategies, and assessing therapeutic response [17,28,29]. For superior clinical decisions, the results should be accessible in 24 to 48 h. In addition, the World Health Organization (WHO) recommends reporting CMV viral load in units of IU/mL and to avoid comparing results between centers or laboratories without assurance or prior documentation of equivalence for testing reagents and processes [28,29]. However, of 201 respondents to a recent survey-based, cross-sectional internet study, only 66 gave thresholds in the units suggested by WHO [32]. 

CMV QNAT may distinguish between the latent virus (low-level CMV DNAemia) and CMV replication (associated with high viral load) [29]. The linearity of the QNAT results in the clinically crucial range between the lower (LLOQ) and upper levels of quantification ought to be demonstrated. Although different tests have different LLOQs, more recent, very sensitive assays have demonstrated LLOQs of less than 200 IU/mL. Very low levels detectable but below the LLOQ may have no therapeutic significance. Because changes in values must be at least twofold (0.5 log10 IU/mL) to signify biologically significant changes in viral replication, QNAT results are evaluated in this manner. QNAT variability is highest for viral loads of 1000 IU/mL and below (3 log10), in which variations may need to be higher than fivefold (0.7 log10 IU/mL) to be considered significant [29]. A comparison of the new and old test performance characteristics must be conducted whenever the laboratory modifies its QNAT or extraction methodologies as well as for following the WHO’s International Reference Standard for assay calibration. In addition, each transplant center should collaborate with their clinical laboratories to establish and validate appropriate center- and assay-specific viral load limits for a range of therapeutic applications [28,29].

The primary disadvantage of CMV QNAT is the lack of universally applicable criteria for various clinical indications. Although implementation of the WHO International Standard for Calibration has significantly increased the level of concordance in viral load measurements across different assays, there is still a clinically significant degree of variation in viral load measurements reported for the same sample when it is tested by various CMV QNAT assays [28,29]. Viral load may vary among WHO-certified assays for a variety of reasons, including variations in assay platform, clinical samples (plasma or whole blood), gene target and amplicon size, and extraction methods, among others [17,28,29]. Moreover, there are certain clinical conditions, such as CMV replication in the transplanted organ without concurrent viremia or the administration of an antiviral that interferes with DNA replication, that may affect measurement of the viral load [17]. 

Compared with the use of CMV NAT, the use of a pp65 antigenemia assay is similar in terms of directing preventive medication, making a rapid and accurate diagnosis of CMV illness, and directing treatment responses. However, the main limitations of this assay are the lack of assay consistency between centers and the requirement to handle the clinical sample in a short period of time (because of the short neutrophil life cycle). In addition, the assay has limited usefulness in SOT recipients with leukopenia since it depends on leucocytes [29]. Hence, the assay is not widely used. A questionnaire-based, cross-sectional online study reported that 97% of the respondents used CMV NAT as a diagnostic tool for CMV, while only 3% use an antigenemia assay [32]. 

The WHO standard for quantitative DNA polymerase chain reaction (PCR) has been used with inadequate knowledge and expertise, indicating a knowledge gap in the understanding and interpretation of viral monitoring laboratory results [32]. Established definitions of CMV detection in blood have been indicated in the CMV Drug Development Forum report to standardize CMV DNA quantification across laboratories and centers. The detection of CMV pp65 antigen in peripheral blood leukocytes is defined as antigenemia. When DNA is detected in samples of plasma, serum, whole blood, or isolated peripheral blood leukocytes or in buffy-coat specimens, it is defined as DNAemia, whereas RNA detection is defined as RNAemia. The isolation of CMV using conventional or rapid culture methods is referred to as viremia. However, current evidence does not support the claim that CMV is replicating in blood simply because the virus, antigen, or DNA is detected there [21].

The diagnosis of end-organ diseases caused by CMV varies according to the infected organ. Along with the detection of CMV in lung tissue, clinical symptoms or signs of pneumonia, such as hypoxia, are required to confirm CMV pneumonia. In suspected cases of CMV hepatitis, abnormal liver function should be tested for. Moreover, central nervous system symptoms are crucial for confirmation of encephalitis. Overall, the identification of histologic features of CMV infection in the involved allograft combined with the detection of CMV by virus isolation, rapid culture, immunohistochemical analysis, in situ hybridization, or quantitative PCR are mandatory to confirm suspected CMV end-organ diseases [21,28,29,30]. However, it can be challenging to recognize the signs and symptoms of CMV end-organ disease, and the assessment of symptoms is frequently subjective and varies by both the patient and healthcare professional [24]. In addition, the invasive procedures used to obtain samples and the availability of less invasive tests have led to a reduction in the use of histopathology tests in CMV diagnosis. However, these tests remain highly recommended when co-pathogens or any concurrent pathology (such acute allograft rejection) is suspected, especially when there is an insufficient response to anti-CMV therapy [29].

## 4. Treatments

The prevention and control of CMV infection are crucial in the medical care of transplant recipients and other immunocompromised individuals [15]. The success of anti-HCMV treatment is influenced by many factors, including diagnostic methods, concentration and duration of antiviral drugs received, and susceptibility of the viral strain to the administered antiviral drug [7,22]. Currently, there are two major preventive approaches following transplantation: “prophylaxis”, which involves giving antiviral drugs to patients who are “at risk”; and “preemptive therapy”, which involves routinely checking the plasma CMV viral load and giving antiviral drugs only when a threshold is exceeded [3,4,8,10,14,33]. The preemptive approach is more common among HSCT recipients unlike SOT recipients for whom the initiation of prophylactic antiviral treatment is the common approach [9,33]. However, it is challenging to use preemptive strategies in centers that perform a large number of transplantations because such approaches require more complicated logistics compared with prophylaxis [13]. The main differences between these two approaches among SOT and HSCT are shown in Table 1 [33]. The antiviral drugs currently administered include ganciclovir, valganciclovir, FOS, cidofovir, brincidofovir (orally available lipid prodrug of cidofovir), maribavir, letermovir, filociclovir (formerly cyclopropavir), leflunomide, and artesunate [7,8,11,12,14]. However, prolonged drug exposure and incomplete suppression of CMV may result in the emergence of drug-resistant mutations [5,11,12]. Furthermore, when a drug to which resistance has developed continues to be administered, it can lead to an accumulation of numerous drug resistance mutations, increased morbidity, and in SOT recipients, shortened graft survival [34]. 

## 5. Drug Resistance

Drug resistance is a consequence of one or more mutations that confer different levels of resistance, with the overall level increasing over time as more mutations occur [5]. A single HCMV with multiple mutations or an infection of combined viruses with various mutations can cause multidrug resistance [11]. Following transplantation, infections with multiple virus strains are common (15–90%) [16]. A recent study analyzing novel drug-resistant HCMV DNA polymerase mutations reported that the co-acquisition of the previously known FOS-resistant mutation T700A and the H600L mutation results in severe resistance to FOS treatment, indicating that T700A and H600L work together to cause FOS resistance. H600L and T700A mutations appear to develop in an individual after prolonged exposure to FOS but are no longer detectable when FOS is replaced with cidofovir, indicating that these mutant viruses are susceptible to cidofovir [11].

Drug resistance should be suspected in a patient with any of the following risk factors: R-/D+ serostatus, receipt of a lung transplant, high pre-treatment CMV viral load, higher intensity of immunosuppression, prolonged subclinical viremia, and exposure to subtherapeutic antiviral drug doses [5,8,12,17,24,34]. When drug resistance is suspected, laboratory testing should be performed and should include the degree of drug resistance and the extent of cross-resistance with other drugs [5,8,35]. This recommendation is because practical changes in treatment regimens are difficult to implement due to the toxicity and logistical challenges associated with alternative drug therapies (life- or sight-threatening disease) [5,35]. However, a recent study reported that CMV genotyping was unavailable or unknown to 54% of the respondents [32]. Still, treatment failure can also be caused by factors other than drug resistance, such as insufficient drug delivery [24]. 

## 6. Diagnosis of Drug Resistance 

Two methods are currently used to diagnose antiviral drug resistance: phenotypic and genotypic assays [5,7,15,36]. Phenotypic assays were the first to be developed, and they have been crucial in identifying and characterizing mutations that occur in target genes as a result of antiviral therapy [5,15]. Although too time-consuming for use in clinical diagnostics, phenotypic assays are still crucial for validating genotypic assays, which are now commonly used to detect drug resistance [5,7]. Genotypic assays take much less time, ranging from a few hours to up to 3 days, and give accurate, affordable, and clinically useful information. Thus, they are often used in the laboratory for a more rapid confirmation of drug resistance [5,36]. However, tests for genotypic resistance have a number of drawbacks, including a lack of standardization, the potential to miss some loci that encode resistance, and the potential to indicate mutations that have not yet been confirmed. Additionally, for the mutant virus to be detectable, the viral load must be higher than a particular threshold, and there must be a particular baseline for the overall viral population. [17]. 

Phenotypic methods are based on the determination of the drug concentration needed to reduce viral growth in cell culture by a specific amount [5,36]. The value of culture-based methods for confirming drug resistance mutations is well known. The “gold standard” for phenotypic drug resistance testing has been considered to be the plaque reduction assay, but the extended time frame of the assay and subjectivity in quantitating infectivity remain limitations. Although alternative methods, such as in situ enzyme-linked immunosorbent assays and DNA-DNA hybridization assays, have reduced the time required for culture of a clinical sample or have enhanced detection of the resistance, none have gained widespread acceptance or are considered a reference standard similar to the plaque reduction assay [5]. Thus, the need for a quicker drug resistance diagnosis led to the development of genotypic techniques for identifying drug resistance mutations [5].

## 7. Genotyping Techniques for Viral Mutation Diagnosis 

Genotypic resistance testing is recommended when a patient has a rising viral load while receiving long-term therapy [5,13,15]. There are two distinct methods for HCMV genotyping: non-PCR-based methods, such as direct restriction enzyme digestion, and PCR-based methods, such as Sanger sequencing and NGS [37,38,39,40,41]. Non-PCR-based methods may fail to detect sequence variability, as they can only detect mutations in specific regions of a genome [5,36,41]. In addition, polymorphisms close to previously identified mutations but not the mutation itself may produce similar-sized fragments of the mutation [5]. By contrast, sequence variability is less likely to be missed by PCR-based methods [36,41]. However, the Sanger sequencing method can only detect nucleotide and amino acid codon changes from the amplified region, and the sensitivity of detection of new variants may be reduced by poor primer design [5,41]. Whole-genome sequencing, on the other hand, has the ability to simultaneously capture all variants and eliminate the need to design and optimize PCR assays, allowing for parallel antiviral resistance testing in a single experiment [41]. However, NGS is not yet widely used in clinical laboratories because it requires specialized knowledge, takes longer to process results than Sanger sequencing, and has interpretive problems that require sophisticated analysis and a bioinformatics pipeline [24,42]. To overcome these difficulties, a recent study established digital PCR methods for the accurate identification of mutations at codons 460, 594, and 595. These three mutations represent 70% of the ganciclovir-resistant clinical isolates. Despite the higher sensitivity and rapidity of digital PCR compared with Sanger and NGS, all three of these approaches can detect only known mutations. However, they can be used to aid in the detection of drug-resistant mutations in the clinical setting [31].

## 8. Sanger Sequencing 

Sanger sequencing is scalable and suitable for handling a normal volume of CMV drug-resistant mutation testing requests in diagnostic laboratories [15]. The approach consists of PCR amplification of regions of the UL97, UL54, and UL56 genes, where the most common and well-known resistance mutations have been described [5,13,15,35,36,41]. The technology is widely available and reasonably well standardized, with a short turnaround time. However, there are some limitations of this technique, including low-quality sequences in the first 15–40 bp that result from primer binding, an inability to distinguish between single base pair differences in segments longer than 900 bp, and the inability to identify mutant subpopulations with abundances below 20% [15,24,35,43]. In addition, the approach frequently fails when viral loads in patient plasma are less than 1000 CMV copies/mL. Moreover, the mixed peaks in Sanger sequence chromatograms may be difficult to interpret [15,34]. 

### 8.1. Targeted Regions

Based on genotypic and phenotypic assays, the reported variant codons can be divided into five general categories. The first category includes variants that do not confer resistance when they are tested phenotypically, whereas the second category represents resistant mutations proven by a recombinant phenotyping method [5]. The last three categories have a spectrum of potential resistance mutations that range from very improbable to highly possible to develop resistance based on their position within the gene coding sequence and the history of drug exposure [5,18]. One category includes mutations that have been reported in baseline sequences or in isolates from drug-sensitive patients; another category includes mutations that have not been reported in baseline sequences but have been detected in isolates from patients who received treatment; and a third category includes mutations that have changed from a known baseline sequence following drug exposure in vivo or in vitro [5]. The loci of current interest are the UL97 gene encoding HCMV kinase, the UL54 gene encoding HCMV DNA polymerase, and the terminase complex encoded by UL56, UL89, and UL51 genes [5,8,15]. However, there will be a need to expand the CMV gene regions covered in diagnostic testing with the growth of our knowledge and the number of approved antiviral drugs [15].

#### 8.1.1. UL97 Kinase

CMV UL97 kinase is responsible for phosphorylating ganciclovir, valganciclovir, filociclovir, and maribavir, a process essential for their antiviral activity [5,12,15]. Given that protein (p)UL97 is crucial for phosphorylating these antiviral drugs, any mutation that affects the protein’s ability to transfer phosphorylation signals will result in ganciclovir-resistance and cross-resistance to any medication that depends on pUL97 for phosphorylation or uses UL97 as a viral target [5,8]. In addition, the degree of residual pUL97 phosphorylation activity and increased drug resistance depends on the position of the mutations [7,8]. However, FOS and cidofovir do not require phosphorylation by UL97. Thus, resistance to FOS and cidofovir currently arises only by mutations of the DNA polymerase gene UL54 [5,7,12]. 

#### 8.1.2. UL54 DNA Polymerase

The UL54 DNA polymerase is the viral target of all currently U.S. FDA-approved medications for CMV therapy [5,15]. There are two main functions associated with this polymerase: nucleotide polymerization and 3′-5′-exonuclease activity (proofreading). The latter function ensures high replication accuracy, which accounts for the low mutation rate [5]. Therefore, UL54 mutations are less frequent and often combine with UL97 mutations that already exist after prolonged therapy, increasing the level of drug resistance [8,15]. However, mutations in the polymerase may increase its efficiency of recognizing and removing the nucleoside analogues of antiviral drugs from the DNA chain. In addition, these mutations may lead to cross-resistance or multidrug resistance by decreasing the affinity for antiviral compounds [7,18]. 

#### 8.1.3. Terminase Complex

The need for additional drug targets, due to the rise of drug resistance, has prompted researchers to expand their study to include more genes, such as the terminase complex, which includes genes UL56, UL89, and UL51. In the first two years of letermovir’s clinical use, numerous case reports of drug resistance and unsuccessful treatment were published. Therefore, the mapping of its main UL56 resistance mutations became necessary. However, in vitro data revealed mutations outside the typical UL56 codon range, in UL89 and UL51 genes. These mutations alone confer only low-grade letermovir resistance but may enhance the effect of other mutations [15]. Recently, Chou et al. conducted genotypic testing for letermovir (LMV) resistance using Sanger sequencing of the CMV terminase gene UL56 in 1165 diagnostic specimens. Their testing results revealed 36 sequence variants among 173 specimens, with LMV resistance mutations detected in 134 specimens. Codon 325 mutations were most common, followed by mutations at codons 369 and V236M. Combinations of some mutations were also found. Their study confirmed that high concentrations of LMV confer increased resistance and suggested that single-step mutations to absolute LMV resistance are an ongoing concern in its therapeutic use [44].

## 9. NGS Platforms

The use of NGS produces enormous amounts of data that are useful for basic and applied research, drug resistance testing, molecular epidemiology of viral pathogens, and the diagnosis of newly emerging viral infections [41,45]. NGS platforms are also widely used in many clinical and research laboratories due in part to their steadily decreasing costs [41,45]. These techniques enable reconstruction of complete HCMV genomes without the need for sequence cloning and allow for learning more about the population variation and coding potential of the virus during infection [41]. However, NGS methods differ in sequencing protocol, throughput, and sequence length as well as in their underlying biochemistry. Thus, prior to purchasing an NGS platform, it is important to carefully assess the diagnostic and research needs of the laboratory [45].

Although the use of NGS has improved HCMV genome sequencing, most NGS-based studies analyzing the entire HCMV genome have used strains isolated in cell culture or amplicons produced by PCR. It is challenging to sequence clinically derived HCMV samples directly because of the low viral load yield during infection [41,46]. Compared with sequences obtained using earlier methods, those obtained using direct sequencing of the HCMV samples are more likely to accurately represent the original viral population [46]. One study reported the feasibility and usefulness of directly detecting HCMV using NGS from a range of diagnostic specimens, offering new perspectives on viral diversity in longitudinally sampled patients [16]. However, various techniques have been coupled with second-generation sequencing platforms to increase the yield of viral load. Most techniques either increase the viral DNA in the sample by multiple sequence displacement amplification or enrich the sample by capturing viral DNA using DNA or RNA probes, a process known as target enrichment. Both techniques depend on additional PCR amplification, which makes them prone to introducing a new sequence bias into the sequencing library [41]. 

Full-length HCMV genomes were first sequenced using Roche 454 pyrosequencing coupled with either or with both Sanger and Illumina sequencing to enhance low-quality regions. However, Illumina sequencing quickly outperformed other platforms due to its improved chemistry, yield, and base quality, producing the majority of the currently published genomes [41]. A previous study designed a nested amplification strategy, using UL97 and UL54 primers, followed by 454 pyrosequencing to enrich viral DNA from clinical specimens with low viral loads. The study included 19 samples with viral loads of 1000 copies/mL of plasma and below, of which 13 samples (68.4%) were amplified successfully. The lowest viral load for which the UL97 and UL54 genes have been successfully amplified was 394 copies/mL of plasma. [34]. Another study used the aforementioned improved technique to compare the abilities of NGS and Sanger sequencing to identify resistant mutations in SOT. Whereas all the drug resistant mutations identified by Sanger were also identified by NGS, six low-abundance resistance mutations, which were present in <20% of the viral population, were identified by NGS. Two of these six mutations were mixed mutations in two patients, which may have significant implications for their treatment. In addition, the detection of mutations by NGS made it possible to distinguish and compare among patients with or without mutations [35]. 

Analyzing HCMV genomes directly from clinical data is now possible through modern methodologies. It is expected that extensive high-throughput sequence data will shed more light on the epidemiology, pathogenesis, and evolution of HCMV in clinical and natural settings. This will make it easier to identify virulence determinants and create new interventions [46]. In 2017, a team of researchers aimed to characterize HCMV genome diversity in immunocompromised patients directly from clinical samples by using the MiSeq system sequencer from Illumina. The sequence reads from different time points were aligned against the consensus sequence from the initial time point to track the dynamics of variants over time. Nearly half of the transplant recipients had a change in the major HCMV genome type. That study’s findings indicate that recurrent HCMV DNAemia after transplantation may not always be the result of the previously replicating strain emerging but, instead, may be the result of other strains emerging that were already present or later acquired and that may have different biological traits. The progression and outcome of HCMV disease may be greatly impacted by this phenomenon [16]. In 2019, the same team published a study investigating HCMV genomic characteristics, including variation, multiple-strain infection, recombination, and gene loss, also using the MiSeq system for sequencing [46]. Both studies from this team used target enrichment to increase the yield of viral load [16,46]. A total of 78 complete sequences with an average size of 235,465 bp (range, 234,316–237,120 bp) and 13 almost-complete HCMV genome sequences were deposited. The analysis of the sequences revealed that single-strain infections were significantly less common in transplant recipients than in congenitally infected patients. In addition, single-nucleotide polymorphism counts in single-strain infections were significantly lower than those in multi-strain infections [46]. 

## 10. Quality Control of Genotypic Assays

When resistance mutations are missed due to insensitive detection, insufficient coverage of viral genetic loci, or failure to call out significant mutations in the primary sequencing data, CMV drug resistance genotyping assays may report false negative results. On the other hand, false-positive mutation readouts, which may lead to unwise switching of therapy, can occur as a result of sample misidentification, contamination, PCR mistakes, or incorrect interpretation of sequencing data [15]. Reliable testing requires adequate CMV DNA content, accurate pre-processing procedures (DNA extraction, enrichment, or amplification), and suitable sequencing of the gene regions having the diagnostic mutations. Turnaround time, logistical complexity, and cost need to be considered and suitable for clinical use [5,15]. 

### 10.1. Sample Requirements

In order to produce a high-quality DNA template required for sequence analysis, the samples must be handled carefully, after which the target DNA has to be extracted [43]. Plasma and whole blood samples are frequently used in genotyping tests [15,24]. It is recommended to use the same sample that is used for the viral load assay [24]. However, as a practical matter, if there is progressive CMV disease while the patient is receiving therapy and no mutations are detected in plasma or blood, obtaining a tissue biopsy or localized fluid (ocular, cerebral spinal fluid, etc.) may be warranted [13,15,24]. Samples with viral loads below 1000–2000 copies or IU/mL are less likely to accurately detect the mutant sequence population as well as determine the complete genome sequence [13,46]. Thus, samples with viral loads above that range are advised [15,16,24,46]. Moreover, a previous study found that the values of library quality measurements vary depending on the type of samples, with urine showing higher values than blood, likely due to a higher ratio of viral to host DNA [46].

### 10.2. Sequencing Technology

#### 10.2.1. Sanger Sequencing 

**Figure 2:**
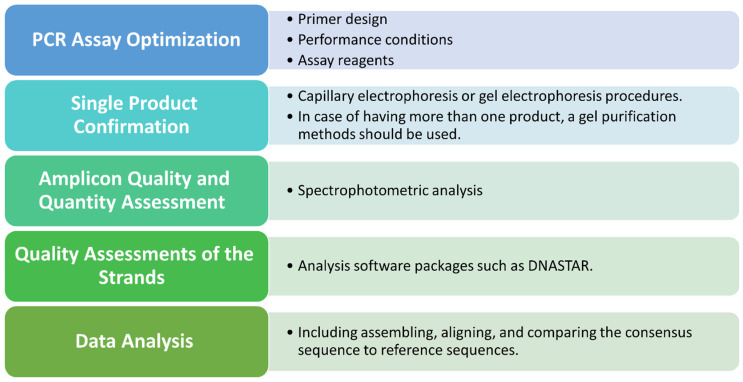
The main considerations of Sanger sequencing performance and analysis. Abbreviations: PCR, polymerase chain reaction.

The standard method involves Sanger sequencing using capillary electrophoresis and an automated system of base calling for processing the chromatogram files of parts of genes in which prevalent resistance mutations have been detected [15]. The key points of Sanger sequencing steps are illustrated in Figure 2. The quality and quantity of targets generated for sequence analysis are affected by the quality of the PCR assay used, including primer design, performance conditions, and optimization of assay reagents. Online open access primer design tools as well as commercial primer design tools are widely accessible and can be used to assist in primer design. These tools ensure a suitable primer length and melting temperature. In addition, they can be used to avoid sequences with a possibility for secondary primer binding, mispriming, and the formation of hairpins or dimers [43]. 

For optimal sequencing results, crucial steps should be taken after PCR amplification. A capillary electrophoresis or gel electrophoresis procedure should be performed to confirm a single product exhibition; otherwise, isolation of the unique band of interest should be conducted using gel purification methods. Following the confirmation of a single product and purification, three essential factors for a successful sequencing reaction should be determined: the concentration of the DNA/DNA amplicon, the length of the amplicon, and the correct amount of primers. In addition, amplicon quantity and quality affect sequencing; thus, they must be assessed either by spectrophotometric analysis using spectrophotometric equipment (e.g., NanoDrop) or ultraviolet fluorescence tagging (e.g., Qubit). However, the purity ratio cannot guarantee successful sequencing, which may also be affected by the location and design of the primers, among other technical sequencing issues [43].

A quality assessment of each strand should be conducted before data analysis. To produce a final sequencing result, it is essential to check the sequence data for accuracy, make any necessary corrections, and then compare the sequence to a reference sequence. Various analysis software packages are available that incorporate sequence quality programs for an initial quality assessment prior to data analysis, such as the DNASTAR genomic suite (https://www.dnastar.com/t-nextgen-seqman-ngen.aspx (accessed on 6 February 2023)), and Geneious (https://geneious.com (accessed on 6 February 2023)). Sequence chromatograms, which can be visualized by the aforementioned software, should be assessed and clarified with a manual review. The unreadable areas, which are typically located adjacent to the primer-binding sites and found in most sequences, should be trimmed from both the forward and complementary strands to ensure a high-quality target sequence. In addition, ambiguous base pairs, which are labeled with the letter “N” rather than being assigned A, G, C, or T, should be clarified by manual assessment of the chromatogram data. In the case of overlapped peaks, if specific binding is detected, they should be interpreted as “mixed sequences detected”; otherwise, the amplification and sequencing steps should be repeated. A case for which the entire sequence should be discarded is the appearance of competing peaks, also referred to as “background noise”, higher than 20% of the main sequence peak. Once the quality assessment is satisfactory, the sequencing data undergo further analyses, which involve assembling, aligning, and comparing the consensus sequence to reference sequences using relevant open access databases [43]. 

#### 10.2.2. NGS

A variety of NGS techniques are offered commercially, and new and improved platforms are constantly being created and released. The underlying biochemistry of these NGS techniques vary, and there are also differences in the sequencing protocols, throughput, and sequence length. For instance, some platforms—such as 454 (Roche Diagnostics, Basel, Switzerland), Illumina sequencing systems, and Ion Torrent (Thermo Fisher Scientific, Waltham, MA, USA)—produce data suitable for de novo assembly, whereas other platforms—such as the SOLiD system (Thermo Fisher Scientific, Waltham, MA, USA))—may be more appropriate for large whole-genome resequencing. Thus, a comprehensive evaluation of the laboratory’s diagnostic and research requirements should be conducted before choosing the technique. The laboratory team should have skills in molecular biology techniques, biotechnology, data management, the use of sequence alignment algorithms, the creation of custom working pipelines, and statistical analysis [45]. 

The following aspects should be considered when choosing an NGS platform for diagnostic virology: diagnostic application, test numbers, cost, speed, accuracy, throughput, read length, ability to upgrade, and automation. For a single application, such as deep sequencing of amplicons, small and cost-effective instruments, such as the Ion Torrent PGM, are available. On the other hand, flexible platforms such as Illumina sequencing systems can handle most diagnostic needs, particularly in situations in which the type and number of tests are not specified. In real-time diagnostics for disease prevention or therapeutic interventions, the main problem for NGS protocols and data analysis is the turnaround time. Therefore, the laboratory ought to develop straightforward protocols for library preparation and bioinformatics tools for simple interpretation and analysis of data [45]. 

## 11. Limitations of Genotypic Testing for Drug Resistance 

A low viral load in a sample or missing testing of a sample from the tissue site where the mutations are localized could lead to false-negative resistance mutation test results. In addition, mutations may be represented as subpopulations that are too small to be detected or that contain genetic loci that have not yet been thoroughly studied. Novel resistance mutations should be confirmed using phenotypic testing, which is complicated by significant standardization and inter-assay issues. In addition, the observed level of resistance may vary because the same mutation may indicate resistance in some assays but not in others [24]. 

## 12. Conclusions 

HCMV is highly pathogenic in immunocompromised patients, such as SOT recipients and allogeneic hematopoietic stem cell transplantation, causing life-threatening disease and representing a major cause of morbidity and mortality [2,3,6,7,8,9,18]. Although several approved antiviral drugs exist, treatment success depends on the diagnostic approaches as well as the concentration, duration, and resistance of the administered antiviral drugs [2,7,15]. Clinical decisions may be facilitated by providing timely information obtained using genotypic testing to detect viral mutations that confer drug resistance [5,15]. The study of these drug resistance mutations is crucial for the development of antiviral drugs as well as basic virological research [5]. The Sanger sequencing of UL97, UL54, and UL56 genes remains the most approachable and scalable option for regular genotypic diagnosis [7,15]. On the one hand, unlike NGS, Sanger sequencing is unable to detect and quantify mutant subpopulations that are less than 20–30% of the population, which may be crucial for determining the most appropriate course of treatment [15,35]. On the other hand, the use of NGS approaches for diagnostic testing requires clinical and analytical validation in accordance with the most recent recommendations and guidelines for molecular assays, and such testing must be supervised by quality assurance and quality control programs [45]. The ideal approach for studying HCMV should involve (i) direct sequencing from clinical samples, (ii) unbiased sequences (either by enrichment or uneven amplification), and (iii) clear evidence of variant co-linearity to a specific viral genome [41]. 

## 13. Future Directions

Despite the abundance of information collected and the advancement of technology, some challenges still exist. For instance, there is no universally recognized definition of reactivation considering how crucial it is to identify subgroups that are at a high risk for reactivation because receiving unnecessary treatment may harm them without providing any additional benefits. In addition, it is unclear whether viremia levels must rise above a certain point to be considered harmful. Thus, the development of an optimized threshold for viral diagnosis is imperative. 

It will become more and more important to create a standardized resistance genotyping database of mutations and incorporate future drugs and drug targets into it as antiviral therapy is used more frequently, and new antiviral medications may be introduced. To be valuable for therapeutic purposes, the database should be regularly updated and available online. Additionally, clinical metadata associated with the viral isolates (such as age, gender, patient cohort, and isolation year) as well as the associated viral information (such as viral load) should be included. This would provide trustworthy data, expand our understanding of the evolution of resistance mutations, and support the predictive power of genotypic testing. 

## Figures and Tables

**Table 1:** The positives and negatives of prophylaxis and preemptive approaches for preventing and controlling HCMV infection in SOT and HSCT recipients.

Prophylaxis			
Positives		Negatives	
SOT	HSCT	SOT	HSCT
Prevents the infection of CMV as well as the disease and indirect effects. Coordination easiness. Large evidence base.	Mortality reduction in recipients with CMV- seropositive.	Antivirals are used in some patients who will not get infected with CMV. Postprophylaxis disease. Neutropenia. High cost	Antivirals are used in some patients who will not get infected with CMV. Ganciclovir/valganciclovir may exacerbate Cytopenia. Acyclovir/valacyclovir’s ineffectiveness. Oral antivirals have a low bioavailability in patients with graft-vs-host disease.
**Preemptive**			
**Positives**		**Negatives**	
**SOT**	**HSCT**	**SOT**	**HSCT**
Limits CMV with delayed onset. Reduce neutropenia occurrence. Simulates natural CMV immunity. Could be more affordable.	Limited toxicity. Accelerates immune system development. Potentially preventing leukemia relapse. Large evidence base.	CMV viral loads in some patients double quickly. Logistical challenges. Unknown impact on CMV indirect effects. Unknown viral thresholds. Unknown testing frequency. Small evidence base.	CMV viral loads in some patients double quickly. Logistical challenges.

Abbreviations: CMV, cytomegalovirus; SOT, solid organ transplant; HSCT, hematopoietic stem cell transplantation.
